# Characterization of *Strip1* Expression in Mouse Cochlear Hair Cells

**DOI:** 10.3389/fgene.2021.625867

**Published:** 2021-04-06

**Authors:** Shasha Zhang, Ying Dong, Ruiying Qiang, Yuan Zhang, Xiaoli Zhang, Yin Chen, Pei Jiang, Xiangyu Ma, Leilei Wu, Jingru Ai, Xia Gao, Pengjun Wang, Jie Chen, Renjie Chai

**Affiliations:** ^1^State Key Laboratory of Bioelectronics, School of Life Sciences and Technology, Jiangsu Province High-Tech Key Laboratory for Bio-Medical Research, Southeast University, Nanjing, China; ^2^Jiangsu Provincial Key Medical Discipline (Laboratory), Department of Otolaryngology Head and Neck Surgery, Affiliated Drum Tower Hospital of Nanjing University Medical School, Nanjing, China; ^3^Department of Otorhinolaryngology, Affiliated Sixth People’s Hospital of Shanghai Jiao Tong University, Shanghai, China; ^4^Co-Innovation Center of Neuroregeneration, Nantong University, Nantong, China; ^5^Institute for Stem Cell and Regeneration, Chinese Academy of Sciences, Beijing, China; ^6^Beijing Key Laboratory of Neural Regeneration and Repair, Capital Medical University, Beijing, China

**Keywords:** hair cell, cochlea, inner ear, expression, *Strip1*

## Abstract

Striatin-interacting protein 1 (*Strip1*) is a core component of the striatin interacting phosphatase and kinase (STRIPAK) complex, which is involved in embryogenesis and development, circadian rhythms, type 2 diabetes, and cancer progression. However, the expression and role of *Strip1* in the mammalian cochlea remains unclear. Here we studied the expression and function of *Strip1* in the mouse cochlea by using *Strip1* knockout mice. We first found that the mRNA and protein expression of *Strip1* increases as mice age starting from postnatal day (P) 3 and reaches its highest expression level at P30 and that the expression of *Strip1* can be detected by immunofluorescent staining starting from P14 only in cochlear HCs, and not in supporting cells (SCs). Next, we crossed *Strip1* heterozygous knockout (*Strip* +/−) mice to obtain *Strip1* homozygous knockout (*Strip1*−/−) mice for studying the role of *Strip1* in cochlear HCs. However, no *Strip1*−/− mice were obtained and the ratio of *Strip* +/− to *Strip1*+/+ mice per litter was about 2:1, which suggested that homozygous *Strip1* knockout is embryonic lethal. We measured hearing function and counted the HC number in P30 and P60 *Strip* +/− mice and found that they had normal hearing ability and HC numbers compared to *Strip1*+/+ mice. Our study suggested that *Strip1* probably play important roles in HC development and maturation, which needs further study in the future.

## Introduction

Cochlear hair cells (HCs) are important cells in the inner ear for receiving sound signals and converting them into electrical signals to be transmitted to the brain (Hudspeth, 2014; Maoileidigh and Ricci, 2019). There are two types of HCs in the cochlea, inner HCs (IHCs) and outer HCs (OHCs), both of which develop in the embryo and mature after birth (Cotanche and Kaiser, 2010; Walters and Zuo, 2013; Fettiplace, 2017). Sensorineural hearing loss is mainly caused by HC loss, which results from many factors such as genetic factors, aging, noise exposure, and aminoglycosides (Dror and Avraham, 2009; Roth et al., 2011). Among these factors, mutations of many important genes involved in the development, maturation, structure, and function of cochlear HCs have been reported to cause HC loss and thus hearing loss (Venkatesh et al., 2015). However, there are still many more genes involved in HC loss that need further study in order to elucidate their roles and mechanisms.

Striatin-interacting protein 1 (*Strip1*), also called FAM40A, is one of the scaffold proteins in the striatin interacting phosphatase and kinase (STRIPAK) complex (Hwang and Pallas, 2014). The STRIPAK complex, an evolutionarily conserved supramolecular complex, is involved in many important physiological processes and diseases, including embryogenesis and development (Lant et al., 2015; Sakuma et al., 2015, 2016; Bazzi et al., 2017; Pal et al., 2017; Zheng et al., 2017), type 2 diabetes (Chursa et al., 2017), and cancer progression (Wong et al., 2014; Zhang et al., 2014; Madsen et al., 2015; Huang et al., 2017; Rodriguez-Cupello et al., 2020). The STRIPAK complex has several different formations depending on the combinations of different, mutually exclusive accessory proteins to the STRIPAK core components (Hwang and Pallas, 2014; Kuck et al., 2019; Rodriguez-Cupello et al., 2020; Seo et al., 2020; Stein et al., 2020; Xie et al., 2020). Several important proteins combine to form the core STRIPAK components, including a striatin family member, the PP2A A/C heterodimer, Mob3, *Strip1* or Strip2, and a GCKIII kinase bound via Ccm3 (Goudreault et al., 2009). Loss of *Strip1* and other scaffolding proteins leads to disassembly and thus to dysfunction of the STRIPAK complex (Madsen et al., 2015; Zheng et al., 2017; Bae et al., 2017; Tang et al., 2019).

The functions of *Strip1* and its homologs in several eukaryotic organisms have been studied previously. In *Neurospora crassa*, the *Strip1* homolog is required for hyphal fusion and cell-to-cell fusion (Xiang et al., 2002; Fu et al., 2011). In yeast, the *Strip1* homolog is important for cell cycle and mitotic progression (Kemp and Sprague Jr., 2003; Frost et al., 2012), and it antagonizes mTORC2 signaling (Pracheil et al., 2012). In *Drosophila*, the *Strip1* homolog regulates circadian rhythms by controlling daytime CLOCK dephosphorylation (Andreazza et al., 2015), plays roles in axon elongation by regulating early endosome organization (Sakuma et al., 2014), and antagonizes Hippo signaling for regulating cell proliferation (Ashton-Beaucage et al., 2014). In *Caenorhabditis elegans*, the *Strip1* homolog is required for endoplasmic reticulum dynamics and function (Maheshwari et al., 2016). Cell migration and cytoskeleton dynamics are regulated by *Strip1* in both *Drosophila* cells and human endothelial cells (Bai et al., 2011; Sakuma et al., 2015, 2016; Suryavanshi et al., 2018). In mouse embryo, knockout of *Strip1* disrupts the migration of the mesoderm in the gastrula stage (Bazzi et al., 2017). *Strip1* is also involved in cancer cell migration and metastasis (Madsen et al., 2015), and loss of *Strip1* results in cell cycle arrest and subsequent reduced tumor growth by inducing the expression of cyclin-dependent kinase inhibitors (Rodriguez-Cupello et al., 2020). Strip2 (also called FAM40B), another Strip proteins, is also one of the scaffold proteins in the STRIPAK complex (Hwang and Pallas, 2014). Strip2 is also reported to play important roles in cell survival, growth, proliferation, differentiation and migration (Sabour et al., 2017; Dai et al., 2019; Pisciottano et al., 2019; Qiu et al., 2020).

In the mouse cochlea, the role of *Strip1* remains unclear, while loss of *Strip2* is reported to lead to a decrease in neural response amplitudes and a reduction in the number of afferent synapses (Pisciottano et al., 2019). Here we first studied the expression pattern of *Strip1* in the cochlea as mice aged from neonates to adults. We then crossed *Strip1* heterozygous knockout (*Strip* +/−) mice to obtain *Strip1* homozygous knockout (*Strip1*−/−) mice but found that *Strip1*−/− mice were embryonic lethal. Finally, we tested the hearing function and counted the HC number in adult *Strip* +/− mice and found no difference compared to wild-type control mice. We suggest that future studies might use *Strip1* conditional knockout mice to study the roles of *Strip1* in cochlear HCs.

## Materials and Methods

### Animals

*Strip* +/− mice, bought from mouse bank of Cyagen company (Stock #KOCMP-229707-*Strip1*, Cyagen), were constructed by deleting exons 3–8 of the *Strip1* gene, and mice of both sexes were used in the experiments. All animal experiments and procedures were approved by the Animal Care and Use Committee of Southeast University. All efforts were made to prevent animals’ suffering and minimize the number of animals used in the experiments.

### Genotyping PCR

Tail tips of mice were used to extract genomic DNA for genotyping PCR. A total volume of 180 μl 50 mM NaOH was added to the tail tips, and these were digested by incubating at 98°C for 1 h prior to adding 20 μl 1 M Tris-HCl (pH 7.0). The primers used in genotyping PCR were as follows: *Strip1*: (F1) 5′-GAC TGG CTG TTT TCC TAC TTA TTC CTA T-3′; (R1) 5′-AGA GCC AGT TCT TTC AAA CGT CAG-3′; and (F2) 5′-GTG GTC TGT TTC CTG AGG ATG TGT-3′.

### RNA Extraction and Real-Time Quantitative PCR

At least 10 cochleae were dissected and used to extract total mRNA with TRIzol (Thermo, #15,596,026), and the mRNA was reverse transcribed into cDNA using the RevertAid First Strand CDNA Synthesis Kit (Thermo, #K1622). Real-time quantitative PCR (qPCR) was performed on a Bio-Rad C1000 Touch thermal cycler using the FastStart Universal SYBR Green Master (ROX) kit (Roche, #4,913,914,001) to quantify the gene expression levels. The real-time qPCR primers were as follows: *Strip1*: (F) 5′-AGT GGA GAA CCA TGC GAC AG-3′, (R) 5′-GGC AAA TGG CTC GTT GTT GT-3′; β*-actin*: (F) 5′-ACG GCC AGG TCA TCA CTA TTG-3′, (R) 5′-AGG GGC CGG ACT CAT CGT A-3′. The relative mRNA expression levels of *Strip1* were calculated using 2^–△△CT^ method relative to the house keeping gene β*-actin*.

### Western Blotting

At least 10 cochleae were dissected and homogenized in 100 μl ice-cold RIPA lysis buffer (Beyotime, #P0013B) using a tissue homogenizer (Shanghai Jingxin Industrial Development Co., Ltd., #JXFSTPRP-48). After centrifuging at 12,000 × *g* for 15 min at 4°C, the supernatant was boiled with 5 × SDS loading buffer, separated by 10% SDS-PAGE, and transferred to an Immobilon PVDF membrane (Millipore, #ISEQ00010). The membrane was first blocked for 1 h at room temperature with 5% non-fat dried milk in 0.1% PBS-Tween 20, incubated with the anti-*Strip1* (Novusbio, # NBP2-45715) and anti-β-actin (Abcam, #ab119716) primary antibodies at 4°C overnight, and then incubated by HRP-conjugated secondary antibodies (Abmart, goat anti-mouse HRP, #M21001 and goat anti-rabbit HRP, #M21002) for 1 h at room temperature. The Supersignal^TM^ West Femto Maximum Sensitivity Substrate (Thermo, #34,094) was used to detect the signals on a FluorChem M system (ProteinSimple, #FM0477).

### Immunofluorescent Staining and Image Acquisition

The cochleae of neonatal mice (P0–P7) were directly dissected in cold HBSS with sharp forceps (WPI), and then fixed for 1 h at room temperature with 4% paraformaldehyde. The cochleae of mice older than P7 were fixed for 1 h at room temperature with 4% paraformaldehyde, decalcified at room temperature with 0.5 M EDTA for 1–3 days (depending on the mouse’s age), and then dissected. After blocking for 1 h at room temperature with blocking solution (5% donkey serum, 1% bovine serum albumin, 0.5% Triton X-100, and 0.02% sodium azide in pH 7.4 PBS), the cochleae were incubated with primary antibodies which are diluted in PBT1 (2.5% donkey serum, 1% bovine serum albumin, 0.1% Triton X-100, and 0.02% sodium azide in pH 7.4 PBS) at 4°C overnight. This was followed by incubation for 1 h at room temperature with Alexa Fluor^TM^ fluorescence-conjugated secondary antibody (Invitrogen) diluted 1:400 in PBT2 (1% bovine serum albumin and 0.1% Triton X-100 in pH 7.4 PBS). Fluorescence mounting medium (DAKO, #S3203) was used to mount the cochleae. The primary antibodies were anti-Myosin7a (Myo7a, Proteus Bioscience, #25-6790, 1:1,000 diluted in PBT1), anti-Sox2 (R&D Systems, #AF2018, 1:400 diluted in PBT1), and anti-*Strip1* (Novusbio, #NBP2-45715, 1:400 diluted in PBT1). A Zeiss microscope (LSM 710) was used to scan the immunofluorescent images with the same hardware settings for all images.

### Cryosections

P30 cochleae were isolated, fixed in 4% paraformaldehyde, and decalcified with 0.5 M EDTA at room temperature as mentioned above. Cochleae were then equilibrated with a series of ascending concentrations of sucrose (10–30%), and treated serially (1:1, 3:7, 9:1, and then 0:1) with a mixture of 30% sucrose and optimum cutting temperature (OCT) compound (Sakura Finetek). During the serial treatment, cochleae were put in each solution for at least 12 h at 4°C, and then vacuumed in a vacuum chamber for 1 h to remove bubbles from the tissue. Tissues were then frozen in OCT, sectioned (10 μm thick) with a freezing microtome (Leica CM1950) and processed for immunofluorecent staining.

### Auditory Brainstem Response (ABR) Test

A total of 100 mg per 1 kg mouse body weight pentobarbital sodium (0.01 g/ml) was intraperitoneally injected into P30 and P60 mice to achieve deep anesthesia, and the closed-field ABR thresholds of the mice were tested by using a TDT System III workstation (Tucker-Davis Technologies) as previously described (Chen et al., 2015). One fine needle electrode was inserted at the cranial vertex, one was inserted underneath the tested ear, and one was inserted in the back near the tail, and the mouse was placed on a thermostatic heating pad in a soundproof chamber. The ABR test was performed by generating 4, 8, 12, 16, 24, and 32 kHz tone pips and the sound intensities were decreased from 90 dB in 10 dB steps.

### Statistical Analysis

Data from at least three independent experiments were statistically analyzed using GraphPad Prism 6 software and are expressed as means± standard errors of the means. Unpaired, two-tailed Student’s *t*-tests were used to calculate *P*-values, and a *P*-value less than 0.05 was considered statistically significant.

## Results

### The Expression of *Strip1* in the Mouse Cochlea Increases as Mice Age

**FIGURE 1:**
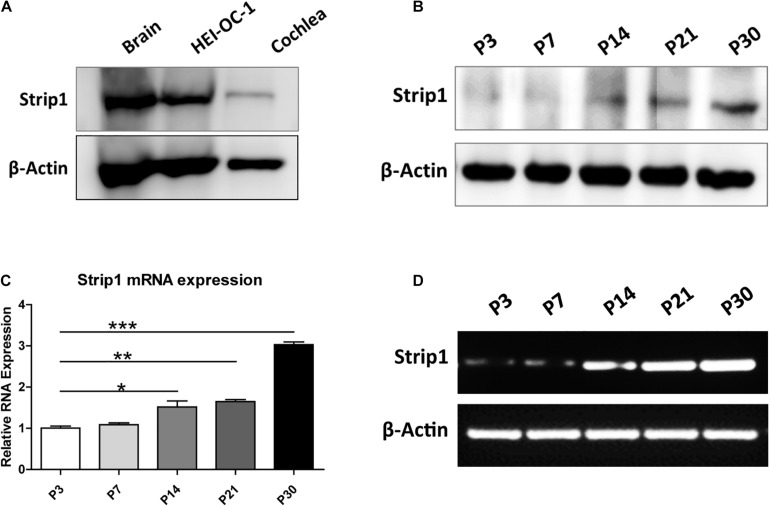
Protein and mRNA expression of *Strip1* in the mouse cochlea. **(A,B)** Western blotting showing the protein expression of *Strip1* in the mouse brain, the HEI-OC1 HC cell line, and in the mouse cochlea at P3 **(A)** and in the mouse cochlea at different ages **(B)**. **(C,D)** mRNA expression of *Strip1* in the mouse cochlea at different ages by qPCR **(C)** and RT-PCR **(D)**. β*-actin* was used as the endogenous reference gene. Four independent qPCR were performed in **(C)**. *p < 0.05, **p < 0.01, ***p < 0.001.

We first studied the protein expression of *Strip1* in the P3 mouse cochlea by western blotting and found that *Strip1* is expressed at a low level in P3 cochlea but at a relatively high level in the HEI-OC1 HC cell line (Figure 1A). Considering that the expression of many genes increases with age, we next studied the expression of *Strip1* at different ages by RT-PCR, qPCR, and western blotting. We found that both the mRNA and protein level of *Strip1* increased as the mice aged and reached its highest level at P30 (Figures 1B–D). These results suggested that *Strip1* might be involved in HC maturation in the mouse cochlea.

### *Strip1* Expression Can Be Detected Only in HCs by Immunofluorescent Staining From P14

**FIGURE 2:**
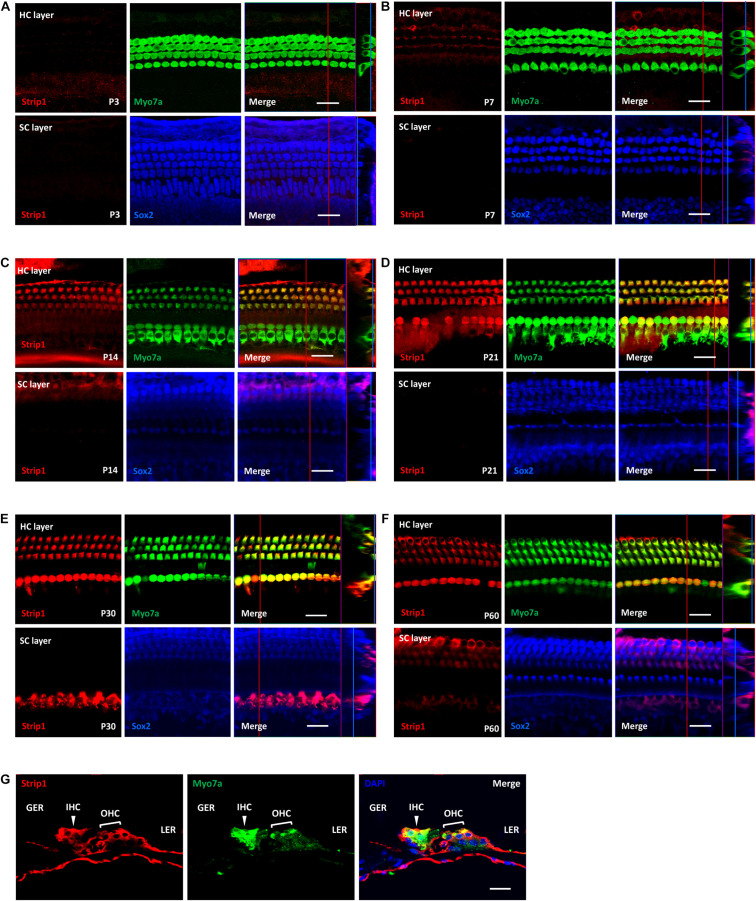
Immunofluorescent staining of *Strip1* in the mouse cochlea at different ages. **(A–F)** The *Strip1* antibody was used to stain *Strip1* in mouse cochleae at P3 **(A)**, P7 **(B)**, P14 **(C)**, P21 **(D)**, P30 **(E)**, and P60 **(F)**. Because nucleus of SCs staining by Sox2 antibody are not always in the same layer, Z projection was performed with the ImageJ software in order to capture all the SCs in the images. **(G)** The immunofluorescent staining of P30 cochlear cryosections also showed the same expression pattern of *Strip1*. Myo7a and Sox2 were used as HC and SC markers, respectively. Scale bar, 20 μm.

Next, we used the *Strip1* antibody to study the specific expression pattern in the mouse cochlea by using immunofluorescent staining. We did not detect any *Strip1* immunofluorescent signal in P3 or P7 cochlea in either the HC layer or SC layer (Figures 2A,B). At P14, we detected obvious *Strip1* immunofluorescence in OHCs, but it was barely detectable in IHCs or the SC layer (Figure 2C). From P21 to P60, there was obvious *Strip1* immunofluorescence in both OHCs and IHCs, but not in the SC layer (Figures 2D–F). We also immuno-stained frozen sections of P30 mice cochlea to confirm the limited expression of *Strip1* in HCs (Figure 2G). These results also showed that *Strip1* expression increased as mice age and were consistent with the mRNA and protein expression of *Strip1*.

### Homozygous *Strip1* Knockout Is Embryonic Lethal

**FIGURE 3:**
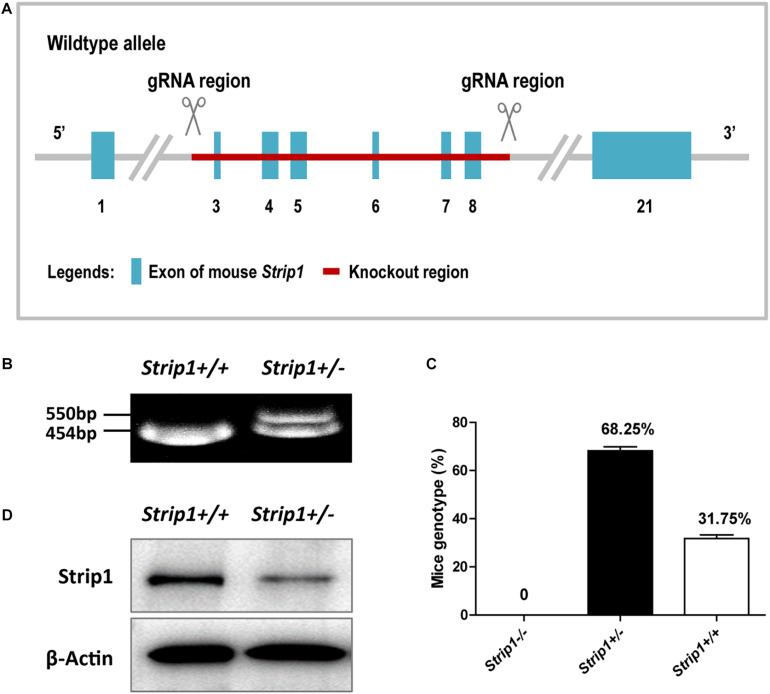
The construction and breeding of the *Strip* +/− mice. **(A)** Exons 3–8 of *Strip1* were deleted to knock out the *Strip1* gene. **(B)** Genotyping of *Strip* +/− mice and their offspring. The mutant allele is 550 bp, and the wild type allele is 454 bp. **(C)** The ratio of mice with different genotypes. There were no *Strip1*–/– mice, while the percentages of *Strip* +/− mice and *Strip1*+/+ mice were 68.25 and 32.75% per litter, respectively. Three litters of mice were counted. **(D)** Western blotting showing *Strip1* protein expression level in *Strip1*+/+ and *Strip* +/− mice. β*-actin* was used as the endogenous reference gene.

Considering the high expression level of *Strip1* in the HCs of the adult mouse cochlea, we speculated that *Strip1* might play important roles in HCs and thus in hearing function in the adult mouse cochlea. Therefore, we constructed *Strip* +/− mice and crossed them to get *Strip1*−/− mice to study the roles of *Strip1* in cochlear HCs (Figures 3A,B). However, genotyping results showed that no *Strip1*−/− mice were born and that *Strip* +/− and *Strip1*+/+ mice were born in an approximately 2:1 ratio (Figure 3C). We also used *Strip1* antibody to observe the protein expression level of *Strip1* in *Strip* +/− and *Strip1*+/+ mice, and found that *Strip1* protein level is decreased in *Strip* +/− mice compared to *Strip1*+/+ mice (Figure 3D). These results indicate that *Strip1*−/− are embryonic lethal, suggesting that *Strip1* plays very important roles during embryo development.

### *Strip* +/− Mice Have Normal Hearing Function and HC Numbers

**FIGURE 4:**
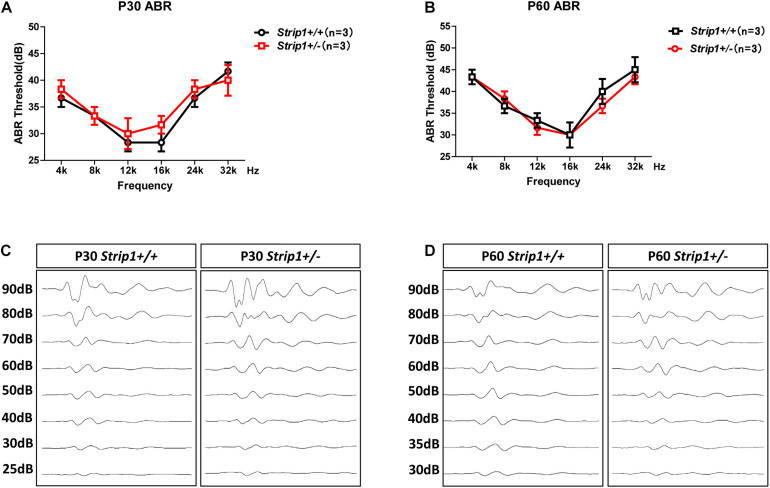
ABR tests of P30 and P60 mice. **(A,B)** The hearing function of P30 **(A)** and P60 **(B)** *Strip* +/− mice and *Strip1*+/+ mice was tested by ABR. n refers to the number of mice. **(C,D)** The ABR waveform at 16 kHz in P30 **(C)** and P60 **(D)** *Strip* +/− mice and *Strip1*+/+ mice.

**FIGURE 5:**
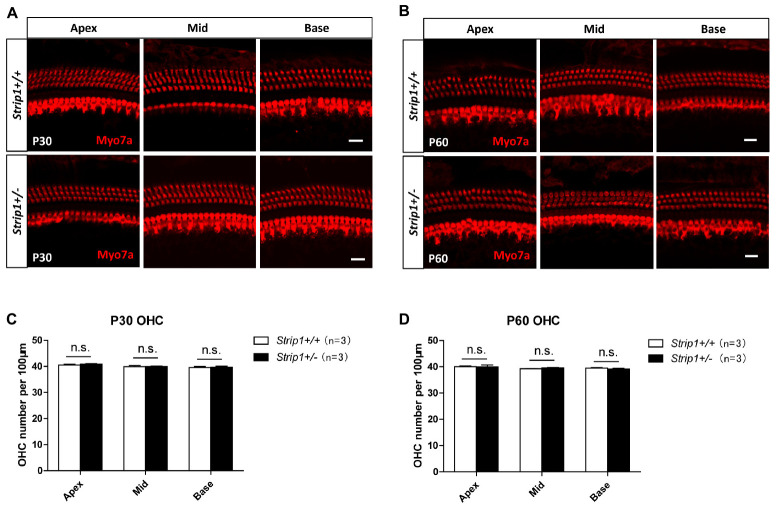
The cochlear HCs in adult *Strip* +/− mice are normal. **(A,B)** Immunofluorescent staining of P30 **(A)** and P60 **(B)** cochleae from *Strip* +/− mice and *Strip1*+/+ mice. Myo7a was used as the HC marker. Scale bar, 20 μm. **(C,D)** Quantification of the OHC number per 100 μm cochlear length in P30 **(C)** and P60 **(D)** *Strip* +/− mice and *Strip1*+/+ mice. n refers to the number of mice. n.s., not significant.

Because we could not obtain *Strip1*−/− mice, we studied the hearing function of *Strip* +/− mice to see whether heterozygous knockout of *Strip1* had any influence on hearing function. ABR tests were performed in P30 and P60 *Strip* +/− mice, and *Strip1*+/+ mice from the same litter were used as controls. The results showed that the hearing function of *Strip* +/− mice was normal at both P30 and P60 compared to the control mice (Figures 4A,B), which suggested that heterozygous knockout of *Strip1* did not disrupt the hearing function of mice. We also examined the waveform of the 16 kHz ABR tests in P30 and P60 mice, and this showed no difference compared to controls (Figures 4C,D). Considering that HCs in the cochlea are necessary for sensing sound vibrations, we examined the HCs of P30 and P60 *Strip* +/− cochleae. Myo7a staining showed that the HCs had similar distributions in *Strip* +/− mice at both P30 and P60 compared to control mice (Figures 5A,B). We also quantified the HC number and found that there was no significant HC loss in *Strip* +/− mice at either P30 or P60 (Figures 5C,D). Taken together, these results suggest that *Strip1* heterozygous knockout does not have any influence on the hearing function or HC number in the adult mouse cochlea. It is possible that only *Strip1* homozygous knockout will have an effect on hearing function and HC number, but because *Strip1*−/− are embryonic lethal, *Strip1* HC conditional knockout mice will be needed to further study the role of *Strip1* in adult mouse cochlear HCs.

## Discussion

HC loss is the main cause of sensorial hearing loss. Although many genes have been reported to be involved in this process, the roles of many other genes remain unknown. Here we found that the expression of *Strip1* in cochlear HCs increases as mice age, which suggests that *Strip1* might play important roles in HC maturation and in maintaining normal hearing function. However, *Strip1*−/− mice die before birth, and we only tested the hearing function and counted the HC number in *Strip* +/− mice, both of which were normal compared to the control mice.

As mentioned above that core STRIPAK components include a striatin family member, the PP2A A/C heterodimer, Mob3, *Strip1* or Strip2, and a GCKIII kinase bound via Ccm3 (Goudreault et al., 2009). Different combinations of these core proteins and accessory proteins results in the formation of different STRIPAK complexes. And it was reported that the STRIPAK complex plays many important roles in embryo development, cancer, diabetes, autism, and many other diseases, and regulates many important signaling pathways and cellular processes (Hwang and Pallas, 2014; Wong et al., 2014; Zhang et al., 2014; Lant et al., 2015; Madsen et al., 2015; Sakuma et al., 2015, 2016; Bazzi et al., 2017; Chursa et al., 2017; Huang et al., 2017; Pal et al., 2017; Zheng et al., 2017; Elramli et al., 2019; Kuck et al., 2019; Tang et al., 2019; Rodriguez-Cupello et al., 2020; Seo et al., 2020; Stein et al., 2020; Xie et al., 2020).

*Strip1* is one of the scaffolding proteins in the STRIPAK complex, which is involved in embryogenesis and development (Lant et al., 2015; Sakuma et al., 2015, 2016; Bazzi et al., 2017; Pal et al., 2017; Zheng et al., 2017). Strip2, another scaffolding protein in the STRIPAK complex, is also reported to play roles in lung adenocarcinoma, smooth muscle, embryonic stem cells, and inner ear HCs (Sabour et al., 2017; Dai et al., 2019; Pisciottano et al., 2019; Qiu et al., 2020). In the cochlea, Strip2 is reported to be expressed in cochlear HCs, and mice lacking *Strip2* have decreased neural response amplitudes and reduced numbers of afferent synapses (Pisciottano et al., 2019). Transcriptome analysis of cochlear IHCs and OHCs from adult mice also identified many differentially expressed genes between IHCs and OHCs, including *Strip2* (Li et al., 2018). Considering the results from these studies, we suspected that *Strip1* might also be expressed and play important roles in the mouse cochlea.

We used real-time qPCR and western blotting to study the mRNA and protein expression of *Strip1* in the mouse cochlea and found that the expression of *Strip1* increased as the mice aged and reached its highest expression level at P30. We also used immunofluorescent staining with a *Strip1* antibody to study the expression pattern of *Strip1* in the mouse cochlea. We found that *Strip1* protein was barely detectable in any HCs at P3 and P7, that it was expressed in OHCs but only barely in IHCs at P14, and that it was expressed in both IHCs and OHCs starting from P21. We did not detect any *Strip1* expression in SCs by immunofluorescent staining. Together, these results showed suggest that *Strip1* is likely to be a very important gene for HC maturation and for normal hearing function.

The *Strip* +/− mice we obtained had knockout of one *Strip1* allele by deleting exons 3–8 of the *Strip1* gene. However, when we crossed *Strip* +/− mice we did not obtain any *Strip1*−/− mice. The *Strip* +/− mice were 68.25% of the litters and the *Strip* +/− mice were 31.75%, and their ratio was about 2:1, which was in accordance with Mendel’s law and suggested that the *Strip1*−/− mice died before birth. A recent study showed that *Strip1* conditional knockout in Sox2-Cre mice experienced arrested development at mid-gestation because of serious disruptions of the mesoderm and its derivatives (Bazzi et al., 2017), and this might be the cause of death of the *Strip1*−/− mice in our study.

Because we could only obtain *Strip* +/− mice, we studied their hearing function and HC number at P30 and P60 and found that their hearing was normal at both P30 and P60 compared to the control mice. We also sacrificed the mice and dissected their cochleae and found that the HC number of *Strip* +/− mice was also normal compared to the control mice. In future studies, we plan to obtain *Strip1*-floxp mice and cross them with Atoh1-Cre mice to conditionally knockout *Strip1* in HCs so as to study the roles of *Strip1* in the mouse cochlea.

It was recently reported that Striatin is required for hearing and affects inner hair cells and ribbon synapses (Nadar-Ponniah et al., 2020). Striatin is found to be specifically expressed in the cell–cell junctions of the inner HCs, which is different from *Strip1* expression as we shown. There are three proteins in the mammalian striatin family, striatin (STRN), S/G2 nuclear autoantigen (STRN3), and zinedin (STRN4) (Benoist et al., 2006). Thus we suspected that *Strip1*, expressed in inner and outer HCs, may interact with other two striatin family proteins which may have similar expression pattern as *Strip1*. This may be the reason why striatin expression is different from *Strip1*. And another Strip protein, Strip2, was also reported to be expressed in HCs (Scheffer et al., 2015; Pisciottano et al., 2019). It is possible that in cochlear HCs, both *Strip1* and Strip2 interact with other two striatin family proteins, not with striatin, which needs further study in the future.

## Data Availability Statement

The original contributions presented in the study are included in the article/supplementary material, further inquiries can be directed to the corresponding author/s.

## Ethics Statement

The animal study was reviewed and approved by the Animal Care and Use Committee of Southeast University.

## Author Contributions

SZ, YD, RQ, YZ, and RC conceived and designed the experiments. SZ, YD, RQ, YZ, XZ, YC, PJ, LW, and JA performed the experiments. XM, XG, PW, and JC analyzed the data. SZ, PW, JC, and RC wrote the manuscript. All authors read and approved the final manuscript.

## Conflict of Interest

The authors declare that the research was conducted in the absence of any commercial or financial relationships that could be construed as a potential conflict of interest.
