# Corrigendum: Characterization of *Strip1* expression in mouse cochlear hair cells

**DOI:** 10.3389/fgene.2024.1538016

**Published:** 2024-12-20

**Authors:** Shasha Zhang, Ying Dong, Ruiying Qiang, Yuan Zhang, Xiaoli Zhang, Yin Chen, Pei Jiang, Xiangyu Ma, Leilei Wu, Jingru Ai, Xia Gao, Pengjun Wang, Jie Chen, Renjie Chai

**Affiliations:** ^1^ State Key Laboratory of Bioelectronics, School of Life Sciences and Technology, Jiangsu Province High-Tech Key Laboratory for Bio-Medical Research, Southeast University, Nanjing, China; ^2^ Jiangsu Provincial Key Medical Discipline (Laboratory), Department of Otolaryngology Head and Neck Surgery, Affiliated Drum Tower Hospital of Nanjing University Medical School, Nanjing, China; ^3^ Department of Otorhinolaryngology, Affiliated Sixth People’s Hospital of Shanghai Jiao Tong University, Shanghai, China; ^4^ Co-Innovation Center of Neuroregeneration, Nantong University, Nantong, China; ^5^ Institute for Stem Cell and Regeneration, Chinese Academy of Sciences, Beijing, China; ^6^ Beijing Key Laboratory of Neural Regeneration and Repair, Capital Medical University, Beijing, China

**Keywords:** hair cell, cochlea, inner ear, expression, *Strip1*

In the published article, there was an error in Figure 5B. The immunofluorescent image of the cochlear apex turn of P60 *Strip1+/−* mice was mistakenly used as the image of the cochlear middle turn in the same group of P60 *Strip1+/−* mice. The correct Figure 5 appears below.

**FIGURE 5:**
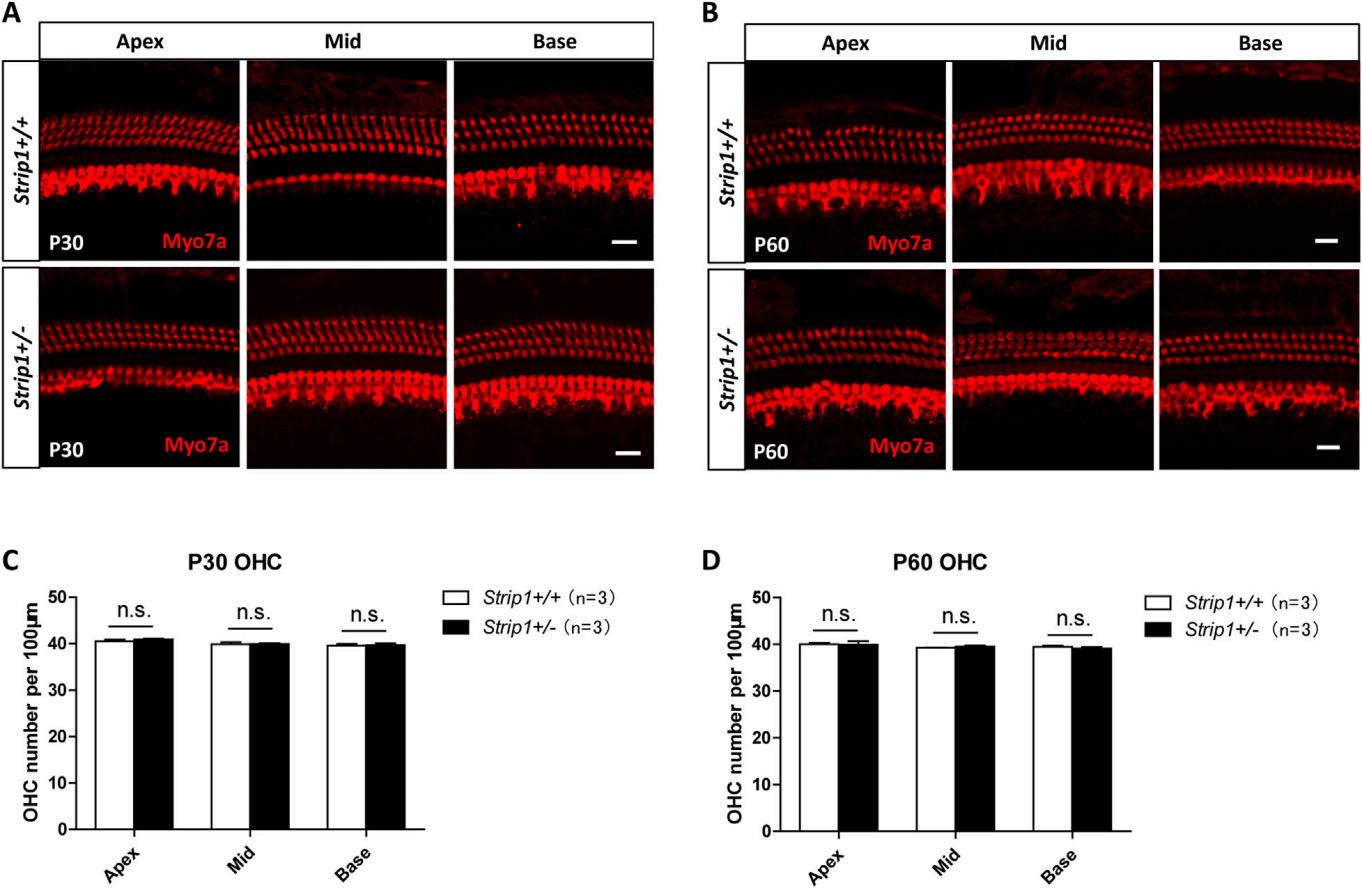
The cochlear HCs in adult *Strip1+/−* mice are normal. **(A,B)** Immunofluorescent staining of P30 **(A)** and P60 **(B)** cochleae from *Strip1+/−* mice and *Strip1+/+* mice. Myo7a was used as the HC marker. Scale bar, 20 µm. **(C,D)** Quantification of the OHC number per 100 µm cochlear length in P30 **(C)** and P60 **(D)** *Strip1+/−* mice and *Strip1+/+* mice. n refers to the number of mice. n.s., not significant.

The authors apologize for this error and state that this does not change the scientific conclusions of the article in any way. The original article has been updated.

